# Tumor-Infiltrating Lymphocytes and Cancer Markers in Osteosarcoma: Influence on Patient Survival

**DOI:** 10.3390/cancers13236075

**Published:** 2021-12-02

**Authors:** José Manuel Casanova, Jani-Sofia Almeida, John David Reith, Luana Madalena Sousa, Ruben Fonseca, Paulo Freitas-Tavares, Manuel Santos-Rosa, Paulo Rodrigues-Santos

**Affiliations:** 1 Tumor Unit of the Locomotor Apparatus (UTAL), University Clinic of Orthopedics, Orthopedics Service, Coimbra Hospital and Universitary Centre (CHUC), 3000-075 Coimbra, Portugal; jmcasanova@fmed.uc.pt (J.M.C.); 8533@chuc.min-saude.pt (R.F.); pftavares@chuc.min-saude.pt (P.F.-T.); 2 Center of Investigation in Environment, Genetics and Oncobiology (CIMAGO), Faculty of Medicine, University of Coimbra, 3000-548 Coimbra, Portugal; jani.almeida@student.uc.pt (J.-S.A.); msrosa@fmed.uc.pt (M.S.-R.); 3 Coimbra Institute for Clinical and Biomedical Research (iCBR), Faculty of Medicine, University of Coimbra, 3000-548 Coimbra, Portugal; 4 Center for Innovation in Biomedicine and Biotechnology (CIBB), University of Coimbra, 3000-548 Coimbra, Portugal; 5 Clinical Academic Centre of Coimbra (CACC), 3000-075 Coimbra, Portugal; 6 Department of Pathology, Robert J. Tomsich Pathology & Laboratory Medicine Institute, Cleveland Clinic, Cleveland, OH 44195, USA; reith@pathology.ufl.edu; 7 Laboratory of Immunology and Oncology, Center for Neuroscience and Cell Biology (CNC), University of Coimbra, 3004-504 Coimbra, Portugal; luana.sousa@student.uc.pt; 8 Institute of Immunology, Faculty of Medicine (FMUC), University of Coimbra, 3004-504 Coimbra, Portugal

**Keywords:** osteosarcoma, tumor-infiltrating lymphocytes, tumor markers, tumor microenvironment, survival

## Abstract

**Simple Summary:**

Osteosarcoma (OST) is the most common type of high-grade primary bone tumor, which mainly affects young adults. Despite the efforts that have been made to address the importance of immune-related factors in OST, there is still a lot to understand. The purpose of the current study was to evaluate the tumor-infiltrating lymphocytes (TIL), the expression of proteins involved in tumor biology, and their impact on the clinical outcome of OST patients. Our results suggest that the presence of tumor-infiltrating CD4+ cells provides protection to patients, and that CD8+ cells have a significant impact on the patient’s overall survival (OS) and progression-free survival (PFS). In addition, a strong association of tumor-infiltrating CD4+ cells and the presence of CD44s expression in tumor samples was observed. These findings reinforce the idea that TIL and the expression of tumor markers should be taken into consideration in order to improve OST treatment and management.

**Abstract:**

Osteosarcoma (OST) is the most common type of high-grade primary bone tumor, which mainly affects young adults. The current standard of care for OST combines surgical resection with chemotherapy. The clinical outcomes and the current options to treat OST patients are unsatisfactory and novel treatment strategies are needed. The crosstalk between tumor cells and immune cells is essential to the OST microenvironment. Despite the efforts that have been made to address the importance of immune-related factors in OST, there is still a lot to understand. The purpose of the current study was to evaluate the tumor-infiltrating lymphocytes (TIL), the expression of proteins involved in tumor biology, and their impact on the clinical outcome of OST patients. We studied 93 samples of OST patients using immunohistochemistry and histomorphometry. We looked for the infiltration of CD3+, CD4+, CD8+, TIA1+ and CD20+ cells and for the expression of CD44 standard (CD44s) and variant 6 (CD44v6), CD95/Fas, Fas-L, p53 and p-glycoprotein. All the parameters were analyzed for the influence on the occurrence of death and metastasis, plus patient overall survival (OS) and progression-free survival (PFS). The effect of sex, age, tumor location (distal femur or proximal tibia) and the combination with neoadjuvant chemotherapy was also assessed. Our results suggest that the presence of tumor-infiltrating CD4+ cells provides protection to OST patients, and that CD8+ cells have a significant impact on the patient’s overall survival (OS) and progression-free survival (PFS), which is more evident in male patients. In addition, a strong association between tumor-infiltrating CD4+ cells and the presence of CD44s expression in tumor samples was observed. Analysis of TIL and tumor markers related to tumor biology could be useful to stratify patients and monitor the response to therapy, as well as to assist with the development of immunotherapy strategies to improve the effects of cytotoxic TIL to eradicate the tumor cells.

## 1. Introduction

Conventional osteosarcoma (OST) is the most common type of high-grade primary bone tumor, which mainly affects young adults [1]. The neoplasm arises preferentially in the metaphyses of the long bones with the highest incidence around the knee [2]. In general, the therapeutic strategy consists of local control surgery, combined with pre- and postoperatively systemic chemotherapy to reduce the likelihood of the disease spreading [3]. For localized OST, the 5-year rate of survival is 70%; however, OST often metastasizes and the 5-year survival rate for recurrent or metastatic disease drastically declines to less than 20% [4,5]. In advanced OST, multidrug resistance to chemotherapy is also a recurrent problem, and the only curative treatment is complete surgical resection of the metastasis, which is a challenging process most of the time [6]. Despite targeted or immune therapies being widely used in various cancers, so far no other strategies have been introduced in OST clinical practice [3]. As such, the clinical outcomes, and the current options to treat OST patients, are unsatisfactory and novel effective treatment strategies are needed. 

OST exhibits high-confidence chromothripsis and a high number of genomic structural alterations, but low tumor mutational burden according to the Pan-Cancer Analysis of Whole Genomes (PCAWG) and others [7,8,9]. Therefore, the use of targeted therapy is limited in this context. On the other hand, the interest in the exploration of novel OST-immunotherapies or immune-related biomarkers to predict the response to therapy has been increasing [10]. Moreover, the osteoid matrix produced by osteosarcoma cells is surrounded by immune cells, amongst blood vessels, mesenchymal and stromal cells. As such, it is evident that immune cells are active players in tumor sustainment and progression [11,12,13], and that the crosstalk between tumor cells and immune cells, through the release of soluble factors and cell–cell contact, is essential for the OST microenvironment [1,14,15,16].

Efforts have been made to address the importance of immune-related factors in OST [10]. Immunological studies have revealed a strong association between the inflammation-based score and the neutrophil–lymphocyte ratio and the overall survival (OS) and progression-free survival (PFS) of OST patients [17,18]. Yet, knowledge about tumor-infiltrating lymphocytes (TIL) and their function in OST is still scarce. Recent studies, mostly using bioinformatics to analyze genome wide expression profiles, correlated the higher infiltration of immune cells with a better clinical outcome [19,20,21,22,23]. Additionally, the potential of prognostic models based on immune-related genes (IRG) profiles or immune scores to predict survival in OST patients has been shown. 

TIL are recruited to the tumor site; however, tumor cells have the capability of escaping the immune response [24]. For example, molecules expressed by tumor cells could input inhibitory signals in immune cells thereby promoting cell death, or redirect an anti-tumor response to a pro-tumoral one, such as in the process of angiogenesis. Given this, the analysis of tumor-associated molecules is essential to better understand the interaction of tumors and immune cells.

In the present study, tumor samples from OST patients were analyzed to identify TIL and to evaluate the expression of molecules related to tumor biology and metastasis. The clinical significance of each parameter compared to patient survival was also assessed. Our data suggest a role for CD4 and CD8 T cells in patient survival, and the expression of CD44s seems to be implicated in CD4 T cell infiltration. The identification of TIL and the evaluation of tumor markers provide valuable information about the tumor’s microenvironment, which can lead to the identification of potential immune-related biomarkers to predict response and improve OST treatment and management. 

## 2. Materials and Methods

### 2.1. Patients and Tissue Samples

A total of 93 OST patients with lesions localized in the distal third of the femur and proximal third of the tibia were identified in accordance with the criteria applied. The specific characteristics of the patients are summarized in Table 1. The inclusion criteria were high-grade osteosarcoma; at stage IIB (G2, T2, M0) according to the Musculoskeletal Tumor Society (MSTS) staging system; lesion localized in the sites stated around the knee; patient age less than 40 years; and treatment initiated by surgical resection or chemotherapy prior to surgery, since both treatments were performed at the same institution. According to these criteria, there were 73 resection specimens without prior chemotherapy, diagnosed and treated by surgical resection (39 cases were from Rizzoli Orthopedic Institute (Bologna, Italy), 20 cases from Rede Sarah (Brasília, Brazil), 7 cases from the Coimbra Hospital and Universitary Centre (Coimbra, Portugal), 7 cases from the Shands Hospital at the University of Florida (Gainesville, FL, USA), and 20 resection specimens from patients diagnosed by surgical biopsy, followed by neoadjuvant chemotherapy, according to previously established protocols, with a minimum of 3 years follow-up (all 20 samples were from Shands Hospital at the University of Florida).

### 2.2. Immunohistochemical (IHC) Staining

The samples studied were obtained from 4 μm thick sections from paraffin-embedded tumor tissue blocks and mounted on poly-L-lysine-coated slides [25,26]. The paraffin present in the mounted sections was melted in the drying oven at 60 °C for 4 h, and the tissue sections were deparaffinized, first in Xylene (2 passages, 5 min each) and then in 95% ETOH (2 min). The slides were immersed in 3% H_2_O_2_ diluted in 95% ETOH for 4 min, to block endogenous peroxidases, and then washed in running water for another 4 min. The primary antibodies used for specific antigen staining and specifications of the antigen retrieval and staining methods for each antigen are summarized in Table S1. Representative images (400× amplification) of each antibody staining are shown in Figure S1.

### 2.3. Histomorphometry

The number of CD3-, CD8-, and CD20-immunereactive cells was quantified by image analysis with a semi-automated system. It was not possible to perform the analysis of CD4- and TIA1-immunoreactive cells. The cell density in each section was measured in a 1 mm^2^ area of tissue. High-resolution images (1315 × 1033 pixels) were acquired using a Zeiss Axiophot microscope (Zeiss, Jena, Germany) with a digital color camera (Diagnostic Instruments, Inc., Sterling Heights, MI, USA). First, the area of the histologic section containing the host–tumor reaction was present. The 1 mm^2^ area of interest was acquired by digital image with the acquisition of four histologic fields (200× amplification). Images were then analyzed with Pro Plus software (Media Cybernetics, Silver Spring, MD, USA).

### 2.4. Data Analysis

The patients were divided according to the presence or absence of each parameter to employ the Log-rank test and plot the Kaplan–Meyer curves to explore the influence of the parameters studied on OS and PFS. Through univariate Cox regression, the individual Hazard Ratio (HR) of each marker for OS and PFS was calculated. The degree of association between the TIL and the proteins studied was estimated using the Chi-square independency test and the quantification of the association was presented by the Phi-coefficient (φ). Phi and Cramer’s V Interpretation were defined as strong (φ > 0.4), moderate (φ > 0.2) and weak (φ > 0.1). Fisher’s exact test was used to analyze the relationship of the presence of TIL and their distribution through the tumoral zone, and the expression of tumor-related markers according to sex, age, tumor localization and the combination of neoadjuvant chemotherapy. The Mann–Whitney U test was employed to compare the cell number between two groups and two-way ANOVA followed by Sidak’s multiple comparisons test was used to compare more than two groups, just as the cell number per region according to the groups studied. Statistical significance was set at *p* ≤ 0.05. ClustVis was accessed online [27] to visualize the clustering of multivariate data using heatmaps [28]. No scaling was applied to rows and imputation was used to estimate missing values. Rows were clustered using correlation distance and average linkage. Statistical tests were performed using IBM SPSS Statistics for Macintosh version 26.0 (IBM Corp, Armonk, NY, USA). The graphical data were executed using Graphpad Prism version 9.1 (GraphPad Software, San Diego, CA, USA).

## 3. Results

### 3.1. Identification of Tumor-Infiltrating Lymphocytes in Osteosarcoma

**Figure 1:**
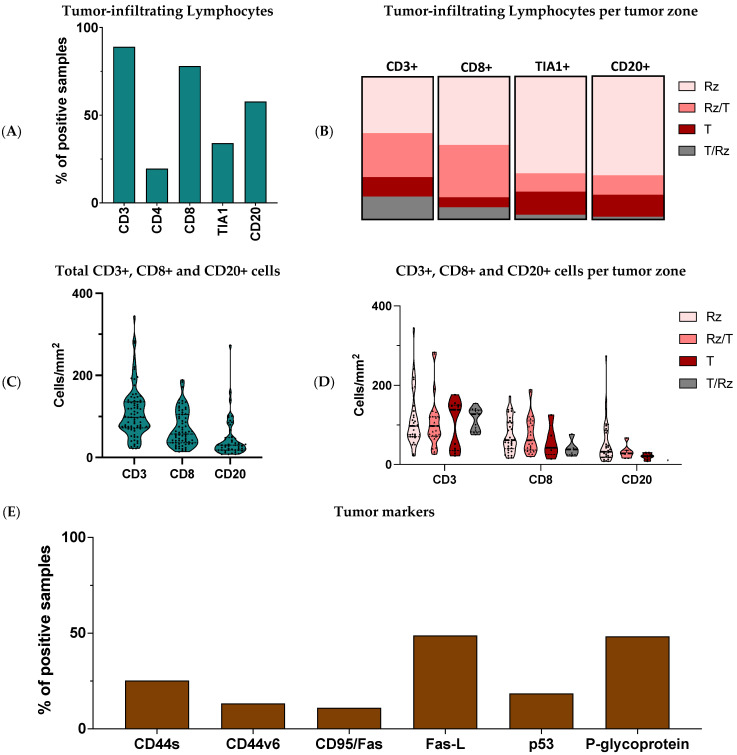
Characterization of the OST microenvironment. Tumor tissues were collected from 93 OST patients and evaluated through IHC staining. (**A**) Frequency of positive samples for CD3+, CD4+, CD8+, TIA1+ and CD20+ infiltration. (**B**) Distribution of the CD3+, CD8+, TIA1+ and CD20+ cells according to the tumoral region. (**C**) CD3+, CD8+ and CD20+ cell number. (**D**) CD3+, CD8+ and CD20+ cell number according to the tumoral region. (**E**) Frequency of positive samples for CD44s, CD44v6, CD95/Fas, Fas-L, p53 and p-glycoprotein expression. Rz—reactive zone; T—tumor zone; IHC—Immunohistochemical.

Immune cells recruited to the tumor site are an active and integrative part of the tumor’s microenvironment. Therefore, we wanted to examine the presence of TIL in the histologic sections of tumor tissue obtained from the 93 OST patients included in the study. Antibodies to CD3, CD4, and CD8 were used to identify T-cell subsets, and antibodies to CD20 were used for B cells. The TIA1 antibody was used to identify cytotoxic lymphocytes (CL). From the IHC analysis, we showed that more than 50% of the samples had inflammatory infiltrates consisting of CD3+ cells (89%, *N* = 81/91), CD8+ cells (78%, *N* = 71/91) and CD20+ cells (57.85%, *N* = 52/90), while infiltration of CD4+ cells (19.60%, *N* = 18/92) and TIA1+ cells (34.10%, *N* = 31/91) was detected in a smaller group (Figure 1A).

Four tumoral histologic zones were identified in the OST samples: (1) the tumor zone (T) corresponding to the trabecular bone containing osteosarcoma; (2) the reactive zone (Rz) related to the local lymphocyte reaction; (3) the interface of the tumor and reactive zones (T/Rz) corresponding most often to the periosteal area; and the interface reactive/tumor zone (Rz/T) containing tumor cells invading surrounding tissues. The infiltrate consisting of CD3+, CD8+, TIA1+, and CD20+ cells was investigated in each of the four zones described above (Figure 1B). In the samples with CD3+ T-lymphocytes (*N* = 81), 39.5% (*N* = 32) were identified in the Rz, 13.6% (*N* = 11) in the T, 30.9% (*N* = 25) in the Rz/T, and 16% (*N* = 13) in the T/Rz. Among the samples with CD8+ T-lymphocytes (*N* = 71), 47.9% (*N* = 34) were identified in the Rz, 7% (*N* = 5) in the T, 8.5% (*N* = 6) in the T/Rz and 36.6% (*N* = 26) in the Rz/T. In the samples with TIA1+ cells (*N* = 31), 67.7% (*N* = 21) were found in the Rz, 16.1% (*N* = 5) in the T, 12.9% (*N* = 4) in the Rz/T and 3.2% (*N* = 1) in the T/Rz. In the samples with CD20+ B-lymphocytes (*N* = 52), 69.2% (*N* = 36) were found in the Rz, 15.4% (*N* = 8) in the T, 13.5% (*N* = 7) in the Rz/T and 1.9% (1/52) in the T/Rz.

The histomorphometry method only allowed the quantification of CD3+, CD8+ and CD20+ cells. Considering the total tissue analyzed, the median cell count obtained per 1 mm^2^ of tissue analyzed was 98 CD3+ cells (Min = 22, Max = 344; *N* = 80), 57 CD8+ cells (Min = 14, Max = 189; *N* = 69) and 30 CD20+ cells (Min = 8, Max = 273; *N* = 51) (Figure 1C). We also determined the average number of CD3+, CD8+ and CD20+ cells according to the tumoral regions (Figure 1D). For CD3, we calculated 113 cells in the Rz (SD = 71, *N* = 31), 99 cells in the T (SD = 62, *N* = 11), 106 cells in the Rz/T (SD = 64, *N* = 25) and 113 cells in the T/Rz (SD = 28, *N* = 13). For CD8, we calculated 73 cells in the Rz (SD = 43, *N* = 34), 58 cells in the T (SD = 43, *N* = 5), 75 cells in the Rz/T (SD = 47, *N* = 25) and 41 cells in the T/Rz (SD = 21, *N* = 5). For CD20, we calculated 54 cells in the Rz (SD = 54, *N* = 35), 21 cells in the T (SD = 8, *N* = 8), 32 cells in the Rz/T (SD = 17, *N* = 7) and 10 cells in the T/Rz (*N* = 1).

### 3.2. Expression of Tumor Markers in Osteosarcoma

The expression of p-glycoprotein, CD44s (CD44 standard), and its CD44v6 isoform (CD44 variant 6), which are associated with tumor biology and the development of metastases, and the expression of the CD95/Fas, Fas-L (Fas ligand) and p53 that are directly involved in cell apoptosis (Figure 1E), were also studied in tumor samples. The expression of CD44s was observed in 25.3% (*N* = 23/91) of the samples, and the expression of CD44v6 in 13.3% (*N* = 12/90) of the samples. P-glycoprotein was present in 48.4% (*N* = 45/93) of the samples. The CD95/Fas was detected in 11% (*N* = 10/91) of the samples; Fas-L was present in 48.9% of the samples (*N* = 44/90); and 18.5% (*N* = 17/92) of the samples exhibited p53 expression.

### 3.3. Tumor-Infiltrating CD4 + Cells—A Protective Factor in Osteosarcoma

To characterize the protective effect of the immune cell populations that infiltrate the tumor, and of the molecules expressed in the tumor cells, we calculated the odds ratio (OR) associated with the occurrence of death (mortality) or metastases for each parameter studied (Table 2). The results obtained revealed a significant association between the infiltration of CD4+ T-lymphocytes and death (*p* = 0.028). The related OR was 0.514 (95% CI: 0.081–0.806), suggesting a decreased risk of death for patients with infiltration of these cells comparatively to those without tumor-infiltrating CD4+ cells. The OR of death calculated for the presence of p-glycoprotein was 2.210 (95% CI: 0.939–5.197). Although this was not statistically significant (*p* = 0.088), the presence of p-glycoprotein expression represented an increased risk of death in the patients studied. No other relevant associations were found.

### 3.4. OS and PFS Correlated with Infiltrating CD8+ T Cells in OST Patients

To understand whether the clinical outcome of OST patients correlated with the parameters analyzed, we considered the time after diagnosis (TAD, months) and the disease-free interval (DFI, months) to calculate the overall survival (OS) and the progression-free survival (PFS), respectively. Overall, the mean estimated TAD was 103 months (95% CI: 85–122) and the mean estimated DFI was 83 months (95% CI: 64–103, Figure S2). For each parameter studied, we analyzed the individual survival curves and calculated the associated hazard ratio; the results are summarized in Table 3.

**Figure 2:**
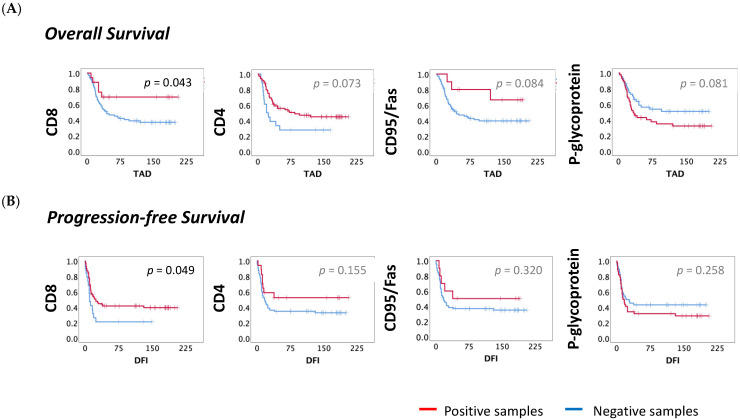
OS and PFS curves according to TILs and the molecules expressed in the tumor. The survival curves for OS and PFS were obtained by the Kaplan–Meyer (Log-rank) method. (**A**) Impact on OS—The median OS for infiltration of CD8+ and CD4+ cells, expression of CD95/Fas and p-glycoprotein non-expression were higher when compared to their counterparts ((CD8−: TAD = 61, *N* = 18; vs. CD8+: TAD = 112, *N* = 69); (CD4−: TAD = 94, *N* = 71; vs. CD4+: TAD = 149, *N* = 17); (CD95/Fas−: TAD = 99, *N* = 77 vs. CD95/Fas+: TAD = 150, *N* = 10); (p-glycoprotein−: TAD = 117, *N* = 45; p-glycoprotein+: TAD = 87, *N* = 44)). (**B**) Impact on PFS—The median PFS for CD8 infiltration was longer when compared to their counterpart (CD8−: DFI = 39, *N* = 19 vs. CD8+: DFI = 91, *N* = 69). IHC—Immunohistochemical; OS—overall survival (months); PFS—progression-free survival; TAD—time after diagnosis (months); DFI—disease-free interval (months). Statistical significance was set at *p* < 0.05.

Focusing on OS, the patients with infiltrating CD8+ cells had a significantly higher estimated TAD compared to those without CD8+ cells (CD8−: 61, *N* = 18; vs. CD8+: 112, *N* = 69; *p* = 0.04), and the risk associated was almost two times lower (HR = 0.526, 95% CI: 0.278–0.993; *p* = 0.048). The analysis of CD4, CD95/Fas and p-glycoprotein demonstrated differences close to the significance level ((CD4−: 94, *N* = 71; vs. CD4+: 149, *N* = 17; *p* = 0.073); (CD95/Fas−: 99, *N* = 77; vs. CD95/Fas+: 150, *N* = 10; *p* = 0.084); (p-glycoprotein−: 117, *N* = 45; vs. p-glycoprotein+: 87, *N* = 44; *p* = 0.081)). Patients with CD4+ cell infiltration or with CD95/Fas expression had a lower probability of death ((CD4+: HR = 0.441, 95% CI: 0.175–1.113, *p* = 0.083); (CD95/Fas: HR = 0.373, 95% CI: 0.116–1.201, *p* = 0.098)). On the other hand, for patients where p-glycoprotein expression was observed, there was a higher chance of death occurrence (p-glycoprotein: HR = 1.638, 95% CI: 0.933–2.875, *p* = 0.086). OS curves for CD8, CD4, CD95/Fas and p-glycoprotein are shown in Figure 2A.

Considering the PFS analysis, the difference of the DFI estimated for patients with or without CD8+ cell infiltration was also statistically significant (CD8−: DFI = 39, *N* = 19 vs. CD8+: DFI = 91, *N* = 69, *p* = 0.049) and those patients with infiltration of CD8+ cells were less likely to develop metastases (HR = 0.561, 95% CI: 0.963–4.498; *p* = 0.062). PFS curve for CD8 is shown in Figure 2B.

Interestingly, when we looked only for patients that had developed metastatic disease, we observed a significant difference in the time of metastasis occurrence in the patients with positive samples for the expression of CD44v6 compared to those without CD44v6 expression (CD44v6−: 15, *N* = 46; vs. CD44v6+: 7, *N* = 8; *p* = 0.048) and those with the expression of CD44v6 develop metastases two times faster (HR = 2.081, 95% CI: 0.963–4.498; *p* = 0.062). The difference between the time to metastasis occurrence of the patients with or without CD44s expression was close to the significance level (CD44s−: 11, *N* = 42; vs. CD44s+: 23, *N* = 13; *p* = 0.088) and patients with expression of CD44s had prolonged PFS (CD44s: HR = 0.561, 95% CI: 0.278–1.134; *p* = 0.107). PFS curves for CD44v6 and CD44s are shown in Figure S3. No other associations with OS or PFS were observed.

OS curves for the location of tumor infiltration of CD3+, CD8+, TIA1+ and CD20+ cells were also examined. Patients with CD3+ cells infiltrated in the T/Rz compared to those with infiltration in Rz had significantly superior TAD (CD3-T/Rz: 137, *N* = 13; vs. Rz: 74, *N* = 31; *p* = 0.025) and DFI (CD3-T/Rz: 119, *N* = 13; vs. Rz: 54, *N* = 31; *p* = 0.037). Patients with CD20+ cells infiltrated in the Rz/T compared to those with infiltration in Rz had significantly superior TAD (CD20-Rz/T: 166, *N* = 7; vs. Rz: 85, *N* = 36; *p* = 0.025) and DFI (CD20-Rz/T: 55, *N* = 7; vs. Rz: 153, *N* = 36; *p* = 0.016). No other differences were found according to region distribution on OS or PFS. We established the cut-off for CD3, CD8, and CD20 cell numbers according to the median cell count (98, 57 and 30 cells, respectively). The survival curves, the risk associated with OS and PFS, and the influence of cell number according to region were evaluated, without significant results.

### 3.5. CD4 T Cells and Tumoral CD44s Expression Were Strongly Associated in OST

**Figure 3:**
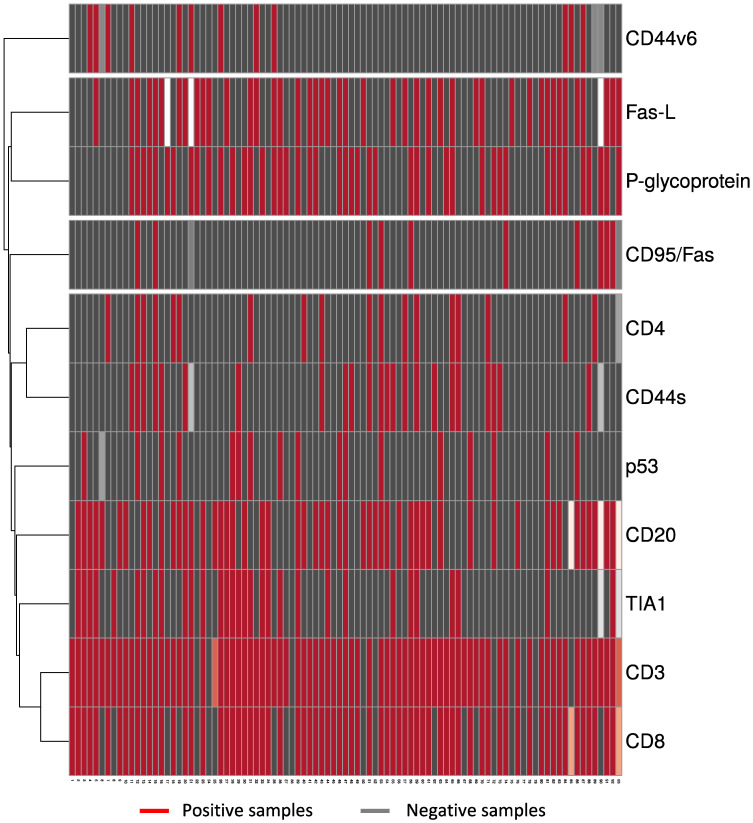
Heatmap of the interactions of TIL and molecules expressed in the tumor. Tumor tissues were collected from 93 OST patients and evaluated using IHC staining. Multivariate cluster analysis was applied to rows for interactions between the presence of CD3+, CD4+, CD8+, TIA1+, and CD20+ cells and the expression of CD44s, CD44v6, CD95/Fas, Fas-L, p53 and p-glycoprotein. ClustVis was accessed online [27] to visualize the clustering of multivariate data using heatmaps. IHC—Immunohistochemical.

To understand whether the infiltrating tumor cells were associated with the expression of the tumor proteins studied, we performed a multivariate cluster analysis using heatmaps and proceeded with a statistical analysis (Figure 3).

From the heatmap we obtained four clusters. The first cluster isolated the expression of CD44v6, the second grouped the expression of p-glycoprotein and Fas-L, the third grouped the expression of CD95/Fas, CD44s and the infiltration of CD4+ cells, the fourth cluster grouped the expression of p53 with the presence of CD20+, TIA1+, CD8+ and CD3+ cells.

Additionally, we quantified the degree of dependency between each cell type that infiltrates the tumor and the molecules studied in samples from OST patients by statistical analysis, the significant and close to the significance level outcomes are summarized in Table 4. We found a strong association between the infiltration of CD4+ cells and the expression of CD44s (φ = 0.408, *p* = 0.000, Table 4) and a moderate association between the infiltration of TIA1+ cells and the presence of p53 (φ = 0.248, *p* = 0.019; Table 4).

**Figure 4:**
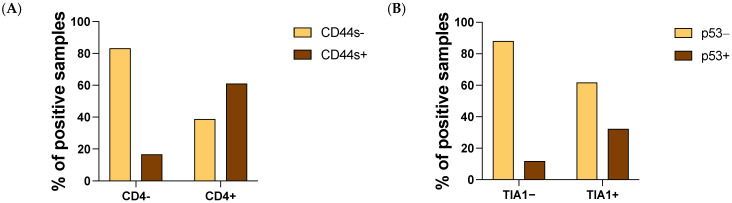
Interaction of CD4 and TIA infiltration with CD44s and p53, respectively, expressed in the tumor. Tumor tissues were collected from 93 OST patients and evaluated using IHC staining. (**A**) Frequency of infiltrating CD4+ cells regarding CD44s tumoral expression. (**B**) Frequency of infiltrating TIA1+ cells regarding p53 expression. IHC—Immunohistochemical.

We observed a higher expression of CD44s and p53 in samples with CD4+ cells and TIA1+ infiltration, respectively (Figure 4A,B).

We further investigated whether the association of the CD4+ cells and the expression of the CD44s molecule was implicated in patient survival. We categorized the patients into four groups: CD4+ and CD44s− samples, CD4+ and CD44s+ samples, CD4− and CD44s+ samples and the remaining CD4− and CD44s− samples. Then, we evaluated the OS and PFS through the Kaplan–Meier curves for each group of patients (Figure S4). Although without significant value, we observed that patients with infiltrating CD4+ cells had longer estimated TAD (CD4+ CD44s−: 144, *N* = 6; vs. CD4+ CD44s+: 143, *N* = 11; vs. CD4− CD44s+: 96, *N* = 11; vs. CD4− CD44s−: 96, *N* = 57; *p* = 0.410) regardless of the expression of CD44s. For PFS no relevant differences were found.

### 3.6. The Influence of Sex, Age, Localization and Neoadjuvant Chemotherapy on the Microenvironment of Tumors in OST Patients

**Figure 5:**
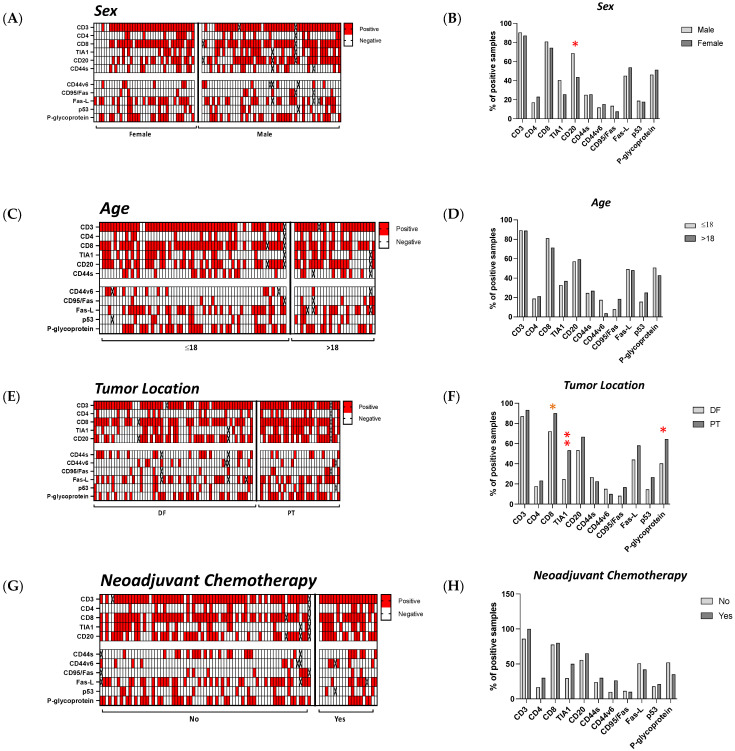
Characterization of the OST microenvironment considering sex, age, tumor location and the use of neoadjuvant chemotherapy. Tumor tissues were collected from 93 OST patients and evaluated using IHC staining. Statistical analysis of the CD3+, CD4+, CD8+, TIA1+, and CD20+ cell infiltration and of the CD44s, CD44v6, CD95/Fas, Fas-L, p53 and p-glycoprotein expression was performed. (**A**) Representative heatmap of the positive and negative samples according to sex. (**B**) Frequency of positive samples in males and females (male: *N* = 54; female: *N* = 39). (**C**) Representative heatmap of positive and negative samples according to age. (**D**) Frequency of positive samples in younger and older patients (≤18: *N* = 65; >18: *N* = 28). (**E**) Representative heatmap of positive and negative samples according to tumor location. (**F**) Frequency of positive samples in patients with the tumor located at the DF or PT (DF: *N* = 62; PT: *N* = 31). (**G**) Representative heatmap of positive and negative samples according to neoadjuvant chemotherapy. (**H**) Frequency of positive samples in patients without any prior treatment to surgery and those that had undergone neoadjuvant chemotherapy (no-NACT: *N* = 73; NACT: *N* = 20). Fisher’s exact test was used for comparative analysis. IHC—Immunohistochemical; DF—distal femur; PT—proximal tibia; *p*-value < 0.05 * or < 0.01 **.

We sought to understand the influence of sex, age, tumor location and neoadjuvant chemotherapy (NACT) on the lymphocyte infiltration and tumor biology. When we analyzed the influence of sex in the group studied, a statistically significant difference was observed for the infiltration of CD20+ cells. Male patients exhibited infiltration of CD20+ cells in 68.6% (*N* = 51) of samples while, for female patients, only 43.6% (*N* = 39) of samples were positive (*p* = 0.020) (Figure 5A,B). The average age of the patients was 18 years, so we compared patients aged less than or equal to 18 years (*N* = 65) with patients over 18 years old (*N* = 28). However, we did not find any statistically significant differences (Figure 5C,D). The tumors included in the study were all found around the knee area, located specifically in the distal femur (DF, *N* = 62) or in the proximal tibia (PT, *N* = 31). Statistical analysis showed significant differences for the infiltration of TIA1+ cells and for the expression of p-glycoprotein (Figure 5E,F). Patients with the tumor located at the PT had more infiltration of TIA1+ cells compared with tumors located in the DF (PT: 53.3%, *N* = 16; vs. DF: 24.6%, *N* = 15; *p* = 0.010) and a higher expression of p-glycoprotein (PT: 64.5%, *N* = 20; vs. DF: 40.3%, *N* = 25; *p* = 0.047). We also found some tendencies for the infiltration of CD8+ cells. Tissue samples from patients located at the PT had more infiltration of CD8+ cells (PT: 90%, *N* = 27; vs. DF: 72.1%, *N* = 44; *p* = 0.063). Among the 93 patients studied, 20 of them were treated with chemotherapy prior to surgery. No significant effect of neoadjuvant chemotherapy was observed in this group of patients (Figure 5G,H).

**Figure 6:**
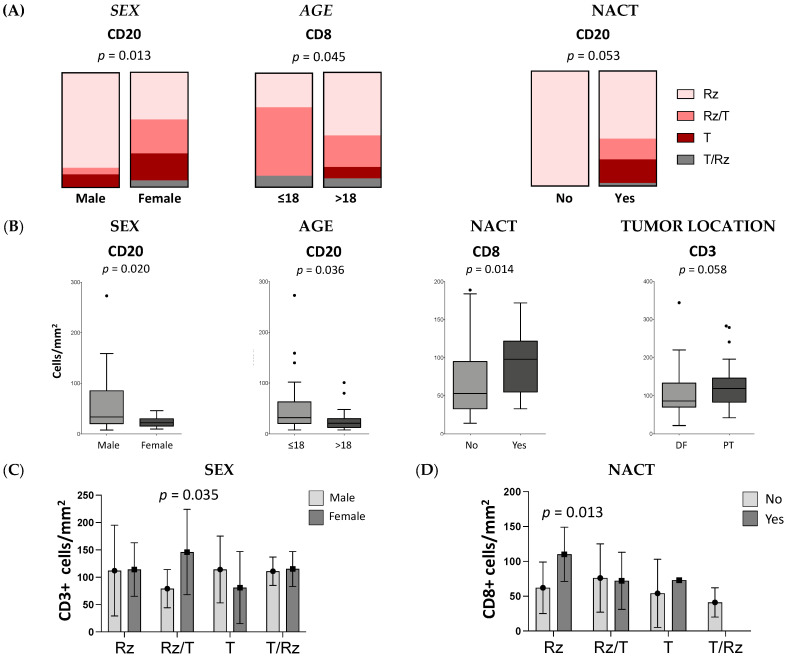
The influence of cell distribution and number on sex, age, tumor location or neoadjuvant chemotherapy. Tumor tissues were collected from 93 OST patients and evaluated using IHC staining. (**A**) Illustration of the tumoral distribution of CD20+ cells by sex, CD8+ cells by age and CD20+ cells by neoadjuvant chemotherapy. Comparative analysis was performed using Fisher’s exact test. (**B**) Count of CD20+ cells according to sex and age, CD3+ cells according to tumor location and CD8+ cells according to neoadjuvant chemotherapy. (**C**) CD3+ cell count per tumoral region in males and females (Rz/T: females = 146, SD = 78, *N* = 10; vs. males = 79 cells, SD = 35, *N* = 15). (**D**) CD8+ cell count per tumoral region in no-NACT and NACT patients (Rz: NACT = 110, SD = 39, *N* = 8; vs. no-NACT = 62, SD = 37, *N* = 26). Mann–Whitney U test was used to compare means between two groups and two-way ANOVA followed by Sidak’s multiple comparisons test was used to compare more than two groups. IHC—Immunohistochemical; DF—distal femur; PT—proximal tibia; NACT—neoadjuvant chemotherapy; Rz—reactive zone; T—tumor zone.

Considering the infiltration sites in the tumor, we analyzed the CD3+, CD8+, TIA1+ and CD20+ cells regarding sex, age, tumor location and neoadjuvant chemotherapy. We found significant differences in the infiltration site of CD20+ cells in males (Rz = 82.9%, Rz/T = 5.7%, T/Rz = 0%, T = 11.4%; *N* = 35) compared with females (Rz = 41.2%, Rz/T = 29.4%, T/Rz = 5.9%, T = 23.5%; *N* = 17, *p* = 0.013) and of CD8+ cells in young patients (Rz = 54.9%, Rz/T = 27.5%, T/Rz = 7.8%, T = 9.8%; *N* = 51) compared with the older patients (Rz = 30%, Rz/T = 60%, T/Rz = 10%, T = 0%; *N* = 20; *p* = 0.045). In samples from patients without treatment prior to surgery, CD20+ cells were identified only in the reactive zone (Rz = 100%, *N* = 13), in contrast to patients subjected to neoadjuvant chemotherapy (Rz = 59%, Rz/T = 17.9%, T/Rz = 2.6%, T = 20.5%; *N* = 39, *p* = 0.053; Figure 6A). According to the groups established, we also observed significant differences in the number of CD20+, CD3+ and CD8+ cells (Figure 6B). On average, male patients had higher numbers of CD20+ cells than females (M: 55 cells, SD = 55, *N* = 34 vs. F: 24, SD = 10, *N* = 17; *p* = 0.020). In addition, younger patients had more infiltrating CD20+ cells than older patients (≤18: 51, SD = 53, *N* = 36 vs. >18: 30, SD = 27, *N* = 15; *p* = 0.036). Tumors located at the PT exhibited a trend for higher infiltration of CD3+ cells (DF: 99, SD = 58, *N* = 53 vs. PT: 127, SD = 66, *N* = 27; *p* = 0.058). The patients exposed to neoadjuvant chemotherapy had a greater number of CD8+ cells than those who were not treated before surgery (no-NACT: 64, SD = 42, *N* = 55 vs. NACT: 93, SD = 41, *N* = 14; *p* = 0.014).

The cell numbers quantified per tumor region were also investigated and some significant differences were found. In the Rz/T we observed higher numbers of CD3+ cells in females compared with male patients (F = 146, SD = 78, *N* = 10; vs. M = 79, SD = 35, *N* = 15; *p* = 0.035; Figure 6C). Likewise, patients treated with neoadjuvant chemotherapy had higher numbers of CD8+ cells localized at the Rz when compared with patients without treatment prior to surgery (no-NACT: 110, SD = 39, *N* = 8; vs. NACT: 62, SD = 37, *N* = 26; *p* = 0.013; Figure 6D).

### 3.7. The Influence of Sex, Age, Localization and Neoadjuvant Chemotherapy on Survival

No influence on OS or PFS was associated with gender (M: 58.1%, *N* = 54; F: 41.9%, *N* = 39), age (≤18: 69.9%, *N* = 65; >18: 30.1%, *N* = 28), tumor localization (DF: 66.7%, *N* = 62; PT: 33.3%, *N* = 31) or neoadjuvant chemotherapy (no-NACT: 78.5%, *N* = 73; NACT: 21.5%, *N* = 20) (Figure S5). We then explored the influence of TILs and the molecules expressed on tumor samples for OS and PFS according to sex, age, tumor location and neoadjuvant chemotherapy.

**Figure 7:**
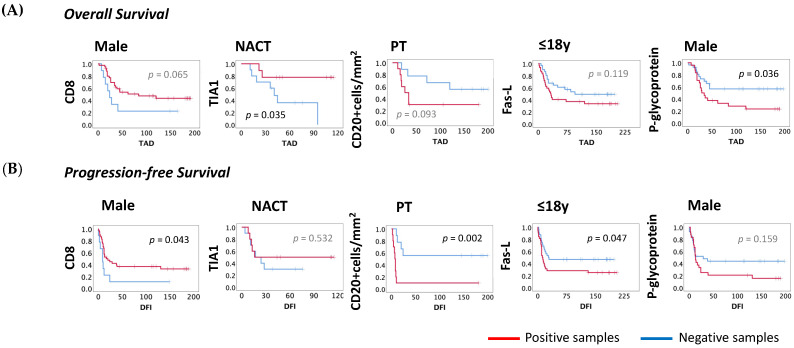
OS and PFS curves of TILs and molecules expressed in the tumor according to sex, age, tumor location and neoadjuvant chemotherapy. The survival curves were obtained by the Kaplan–Meyer (Log-rank) method. (**A**) Impact on OS—In male patients, the OS for p-glycoprotein non-expression was higher when compared to their counterpart (*p*-glycoprotein−: TAD = 123.477, *N* = 27; vs. p-glycoprotein+: TAD = 71.788, *N* = 24). In addition, in males the PFS for CD8+ cell infiltration was higher compared with their counterpart (CD8−: DFI = 24, *N* = 9; vs. CD8+: DFI = 76, *N* = 40). (**B**) Impact on PFS—In younger patients, the PFS for FasL expression was lower compared with their counterpart (Fas-L−: DFI = 100, *N* = 32; vs. Fas-L+: DFI = 63, *N* = 32). In NACT patients, the OS for TIA1+ cell infiltration was higher compared with the absence of infiltration (TIA1−: TAD = 52, *N* = 10; vs. TIA1+: TAD = 95, *N* = 9). In patients with tumors located in the PT region, the PFS for the cell number of infiltrating CD20+ cells were higher compared with their counterpart (CD20−: 118, *N* = 9; vs. CD20+: 24, *N* = 10). IHC—Immunohistochemical; OS–overall survival (months); PFS—progression-free survival; TAD—time after diagnosis (months); DFI—disease-free interval (months); NeoChem—Patients treated with neoadjuvant chemotherapy. Statistical significance was set at *p* < 0.05.

In male patients, we observed lower TAD for those with positive samples for p-glycoprotein (p-glycoprotein-: 123, *N* = 27; vs. p-glycoprotein+: 72, *N* = 24; *p* = 0.036), with approximately two times greater risk of mortality (HR = 2.175, 95% CI: 1.025–4.616; *p* = 0.043; Figure 6). In addition, in males the CD8+ cell infiltration was associated with a higher DFI (CD8−: 24, *N* = 9; vs. CD8+: 76, *N* = 40; *p* = 0.043) and a lower risk of developing metastasis (HR = 0.285, 95% CI: 0.249–1.506; *p* = 0.056; Figure 7A,B).

Considering the patient’s age, no statistically significant differences were found regarding patients’ OS. However, for DFI, we observed a statistical difference for the expression of Fas-L in the group of younger patients (Fas-L−: 100, *N* = 32; vs. Fas-L+: 63, *N* = 32; *p* = 0.047) and those with Fas-L positive expression were almost two times more likely to develop metastases (HR = 1.847, 95% CI: 0.989–3.448; *p* = 0.054; Figure 7A,B).

In tumors located in the PT no significant differences were observed regarding TILs or the expression of cancer markers. Considering patients who underwent NACT, longer TAD was associated with the infiltration of TIA1+ cells (TIA1−: 52, *N* = 10; vs. TIA1+: 94, *N* = 9; *p* = 0.035) and with a lower risk of death (HR = 0.206, CI: 0.041–1.027; *p* = 0.054; Figure 7A,B).

The influence of CD3, CD8 and CD20 cell numbers on OS and PFS was also considered. We observed that, in patients with the tumor located in the PT zone, higher numbers of CD20+ cells correlated with longer DFIs (CD20−: 118, *N* = 9; vs. CD20+: 24, *N* = 10; *p* = 0.002), and the risk of these patients’ developing metastases was significantly lower (HR = 6.171, 95% CI: 1.770–21.510; *p* = 0.004). No other effect on OS or PFS was observed for the cell number quantified in each group analyzed (Figure 7A,B).

## 4. Discussion

In the present study, we sought to explore the infiltration of TIL and the expression of tumor markers implicated in tumor biology, and their influence on OS and PFS in OST patients.

It is well known that immune cells are active elements of the tumor microenvironment that support the development and growth of the OST [12]. Our data showed infiltration of T (CD3+), CD8 T (CD8+) and B cells (CD20+) in more than 50% of the tumor samples and a lower infiltration of CD4 T cells (CD4+) and CL (TIA1+). Like others, we concluded that TIL are recruited to the tumor site suggesting that OST tumors are susceptible to the anti-tumoral activity of TIL, which controls, at least in part, the aggressiveness of the tumor [29,30]. In the case of the OST of the jaws, it has been suggested that the absent or weak infiltration of CD4 and CD8 T cells was one of the possible explanations for the aggressiveness of this specific type of bone tumor [31]. Recent studies have shown that tumor-infiltrating immune cells play an important role in the progression of OS. Thus, we investigated whether there was a risk or protective factor for death, or the occurrence of metastases associated with the immune cells that infiltrate the tumor.

A study centered on immune cells and gene signatures in OST revealed that CD4+ T cells may represent a potential prognostic factor, and suggested that specific analysis of the signature of Th1 cells may improve prognosis in OST patients [32]. Interestingly, even though only 20% of the samples studied had CD4+ T cell infiltrates, our results corroborate the previous work suggesting that infiltrating CD4+ cells give protection from death to OST patients. Nonetheless, a recent study in OST [33] showed a higher proportion of CD4 T cells regarding the infiltration of CD8+ cells. In our group of patients, we found a reduced number of CD4+ cells compared with CD8+ cells, and this difference may be due to the different techniques employed, namely that the results obtained through immunohistochemistry and mass cytometry were difficult to compare. No other parameter studied showed significant risk or protection associated with mortality or metastatic disease.

Beyond the statistical odds for mortality or occurrence of metastases in OST patients, we also evaluated the OS and PFS according to the presence or absence of each TIL and calculated the individual, associated HR. In our study, besides the risk associated to the absence of infiltrating CD4+ T cells we did not find a significant statistical value for OS, even though the impact of CD4+ cells on OS was close to the significancy level, which suggests that CD4+ cells may have some clinically significant value in OST.

We also observed that higher infiltration of CD8+ cells conferred superior OS and PFS in the patients studied, suggesting a possible indicator of a good response. Our results were in line with what has already been reported, namely the importance of infiltrating CD8 T cells on OST patient survival [30,34]. A recent study on OST established a prognostic model based on the expression of 14 immune-related genes (IRG) that stratifies patients in low or high risk for the occurrence of metastases, and the score risk was negatively correlated with infiltrating CD8 T cells and macrophages, and positively correlated with B cell infiltration [20]. Another study identified two gene-immune subtypes of OST based on high and low expression of IRG using bioinformatics analysis [22]. The highest infiltration of CD8+ T cells and anti-tumoral macrophages were observed in the high IRG expression subtype and those patients had a better prognosis than patients in the low IRG expression subtype. Another model to stratify OST patients based on IRG showed a positive relationship between high-level CD8+ cell infiltration and good responders [23]. In patients with the tumor located at the PT, we found a tendency for a higher infiltration of CD8+ cells compared to DF tumors. This suggests that patients with the tumor located at the PT are more likely to achieve a longer survival. The distribution of CD8+ cells according to the four tumor zones defined was different in the age groups. In older patients, CD8+ cells did not infiltrate the tumor and remained in the adjacent areas, unlike younger patients. We also had a greater number of infiltrating CD8+ cells in patients undergoing neoadjuvant chemotherapy, namely at the Rz. When we examined the influence on patient survival, we observed that the influence of CD8+ cells on PFS was more evident in male patients. Together, these findings suggest an increased benefit for: patients with tumors in the PT area, for younger patients, for patients who received neoadjuvant chemotherapy and for male patients regarding CD8+ cell infiltration.

B cell infiltrates were identified in tumor samples from OST patients [13]; however, studies centered on B cells are scarce. We found expression of CD20+ cells in half of the OST samples studied and we did not find significant associations with patient OS and PFS. Nevertheless, we found a lower infiltration in female patients and the distribution in the tumor zone was also different from male patients. A lower infiltration in the oldest patients was also observed and, in patients with the tumor located at the PT, a higher infiltration was correlated with inferior PFS. The above-mentioned study that established an IRG-based prognostic model to stratify OST patients correlated the score risk with the infiltration of B cells positively [20].

The study of molecules expressed by the tumors provides important information about the behavior of the tumor itself. Given this, the expression of molecules related to apoptosis, drug resistance and metastatic capacity were evaluated. We found that approximately 50% of the OST samples had p-glycoprotein and Fas-L expression, while lower percentages were observed for CD44s, CD44v6, CD95/Fas and p53.

P-glycoprotein is a drug efflux pump and its overexpression by tumor cells has been implicated in multidrug resistance in several solid tumors [35]. In OST, p-glycoprotein has been associated with resistance to chemotherapy and it has been hypothesized that targeted therapy to p-glycoprotein could be of importance to overcome the chemotherapy resistance in OST [36,37,38]. When we explored the risk or protective value of the presence of each tumor marker, we observed that patients with p-glycoprotein expression had a higher chance of death occurrence, despite only presenting a statistical value close to the significance level. Similarly, when we analyzed the OS according to p-glycoprotein expression, we observed a lower survival for those with p-glycoprotein expression. In the literature, the predictive value of this protein is still controversial [26,39,40]. The analysis of p-glycoprotein according to the tumor location was different. The percentage of positive samples for p-glycoprotein expression was higher in PT patients but did not correlate with patient survival or the occurrence of metastases. On the other hand, the expression of p-glycoprotein was significantly associated with a decreased OS rate in male patients.

Fas-L-induced apoptosis upon engagement with its own receptor (CD95/Fas) contributes to immune cell-mediated cytotoxicity [41]. In OST, the increased expression of CD95/Fas has been correlated with a reduced metastatic potential of tumor cells [42,43]. As expected, in our study, a trend towards longer survival was observed in patients with CD95/Fas expression. In OST, the expression of Fas-L has been implicated in the occurrence of metastasis [44,45]. Overall, we did not observe a predictive value for Fas-L expression; however, the expression of Fas-L was correlated with poor survival in the youngest group of patients. The manipulation of this pathway could be another strategy to improve OST treatment.

Additionally, we investigated whether there was a significant association between TILs and the molecules studied. To our knowledge, for the first time in the field, we found a strong relationship between the infiltrating CD4+ cells and CD44s expression. CD44 is a homing cellular adhesion protein that could be expressed in a standard form (CD44s) as well as a multitude of CD44 variants (CD44v) [46]. CD44s is known to exert recruitment functions in hematopoietic cells [47]. CD44v has been associated with the ability of tumor cells to adhere to, migrate to and invade other body sites [48]. However, the role of the link between CD44s expression and CD4+ cells is still unknown. Several authors have identified the expression of CD44 and its isoforms in tumor tissue from OST patients, which has been mentioned as a key factor in metastasis development and drug resistance [49,50,51]. Particularly, the expression of the CD44v6 isoform has been associated with the occurrence of metastasis [51,52]. Our data revealed that the time for metastasis to occur is two times faster in patients with CD44v6 expression. Interestingly, the survival analysis for CD44s revealed that for patients where metastasis occurred, the time to metastasis was longer for patients with CD44s expression. We found a strong association of tumor-infiltrating CD4+ cells and the expression of CD44s and further evaluated the survival rate of patients with positive or negative samples for each factor. Although without statistical value, we observed higher TAD for those with CD4+ cell infiltration independent of the CD44s expression. The presence of CD44s expression without CD4+ cell infiltration revealed lower TAD, similar to the patients with negative samples for both. No significantly different TAD was observed in patients with samples positive for both CD4 and CD44s when compared to those with positive samples only for CD4+ cell infiltration. We hypothesized that CD44s expression may be involved in CD4+ cells recruitment, and that the presence of CD44v6 expression may represent a defense mechanism of host cells.

Despite no associations being found between TIA-1 and p53 expression and disease progression in our study group, a moderate positive association was observed for the infiltration of TIA-1+ cells and for the expression of p53. TIA-1 was described as a cytotoxic granule-associated protein that is expressed on cytotoxic lymphocytes as NK and CD8 T cells [53]. In localized OST, the infiltration of cells positive for CD8 and TIA-1 at diagnosis was correlated with a superior survival rate [54]. The p53 protein is encoded by a tumor suppressor gene and the deficient function of p53 affects the bone microenvironment, therefore opening doors for the development of the OST [55,56]. Regarding TIA-1+ infiltrates we found that patients with the tumor located in the PT were more susceptible to TIA-1+ cells and the infiltration of these cells conferred superior survival to patients that were treated with neoadjuvant chemotherapy.

## 5. Conclusions

The major findings of this study were the protective character of infiltrating CD4 T cells, the influence of CD8 T cells on survival and the strong association between the infiltrated CD4 T cells and the CD44s expression. This study confirmed the importance of studying the cells that are able to be recruited and exert their function against tumor cells in the context of OST. The expression of tumor markers is also a matter to be considered and should be further explored to bring new insights about the mechanisms that promote the tumor’s growth and escape. Thus, the identification of novel targets or biomarkers with a predictive value that may be useful to stratify patients and monitor the response to therapy could occur, as well as assisting with the development of immunotherapy strategies to improve the effects of cytotoxic TIL on the eradication of tumor cells.

## Figures and Tables

**Table 1:** Clinicopathological features from OST cases included in the study.

Characteristic, Unit	*N* (%)	Mean (95% CI)
Samples	93	
Sex (proportion female)	39 (41.9%)	
Age, years		18 (17–21)
≤18	65 (70.0%)	
>18	28 (30.0%)	
Localization		
Distal Femur	62 (66.7%)	
Proximal Tibia	31 (33.3%)	
Neoadjuvant Chemotherapy	20 (21.5%)	
Metastasis	57 (61.3%)	
TAD, months		103 (85–122)
DFI, months		83 (64–103)

Legend: OST—osteosarcoma; TAD—time after diagnosis; DFI—disease-free interval; CI—Confidence interval.

**Table 2:** Descriptive statistics of the calculated relative risk of death or metastasis occurrence associated with the presence of CD3+, CD4+, CD8+, TIA1+ and CD20+, or with the expression of CD44s, CD44v6, CD95/Fas, Fas-L, p53 and p-glycoprotein.

Markers		Mortality			Metastization		
		Fisher’s Exact Test	OR	95% CI	Fisher’s Exact Test	OR	95% CI
**Tumor-Infiltrating** **Lymphocytes**	**CD3**	0.503	0.514	0.124–2.138	0.734	0.623	0.150–2.587
	**CD4**	**0.028**	**0.256**	**0.081–0.806**	0.177	0.433	0.152–1.232
	**CD8**	0.183	0.420	0.135–1.305	0.199	0.456	0.149–1.391
	**TIA1**	0.656	0.812	0.332–1.986	1.000	1.056	0.434–2.566
	**CD20**	1.000	0.995	0.417–2.377	0.385	1.529	0.649–3.604
**Tumor** **Markers**	**CD44s**	0.226	0.526	0.201–1.380	0.806	0.805	0.309–2.099
	**CD44v6**	1.000	1.190	0.346–4.093	0.756	1.391	0.386–5.015
	**CD95/Fas**	0.105	0.305	0.073–1.269	0.509	0.620	0.166–2.136
	**Fas-L**	0.386	1.602	0.681–3.768	0.289	1.624	0.693–3.806
	**p53**	0.163	0.405	0.133–1.236	0.271	0.500	0.173–1.447
	**P-glycoprotein**	0.088	2.210	0.939–5.197	0.201	1.874	0.802–4.379

Legend: Row cells with dark background represent statistically significant value and cells with light background represent statistical value close to the significance level. OR—odds ratio; CI—Confidence Interval.

**Table 3:** Descriptive statistics of the overall survival, progression-free survival and associated risks calculated for all the parameters evaluated in the present study.

Markers		Overall Survival				Progression-Free Survival			
		Log-Rank Test	TAD (Months)		HR Exp (B) (pos)	Log-Rank Test	DFI (Months)		HR Exp (B) (pos)
			neg	pos			neg	pos	
**Tumor-Infiltrating** **Lymphocytes**	**CD3**	0.201	74	107	0.60	0.414	66	87	0.723
	**CD4**	0.073	94	149	0.441	0.744	75	114	0.590
	**CD8**	**0.043**	**61**	**112**	**0.526**	**0.049**	**39**	**91**	**0.561**
	**TIA1**	0.794	103	108	0.922	0.631	85	81	1.143
	**CD20**	0.773	103	103	0.919	0.678	89	77	1.120
**Tumor** **Markers**	**CD44s**	0.361	100	115	0.725	0.369	80	92	0.756
	**CD44v6**	0.389	106	94	1.419	0.368	86	73	1.403
	**CD95/Fas**	0.084	99	150	0.373	0.320	81	104	0.634
	**Fas-L**	0.154	115	94	1.513	0.158	93	75	1.459
	**p53**	0.200	98	127	0.577	0.299	76	105	0.678
	**P-glycoprotein**	0.081	117	87	1.638	0.258	93	71	1.344

Legend: Row cells with dark background represent statistically significant value and cells with light background represent statistical value close to the significance level. TAD—time after diagnosis; DFI—disease-free interval; HR—Hazard Ratio; neg—negative samples; pos—positive samples.

**Table 4:** Descriptive analysis of the degree of association between the presence of TIL and the expression of molecules related to tumor biology.

TIL	Molecule	Pearson Chi-Square	Phi Coefficient	Effect Size
CD4	CD44s	0.000	0.408	Strong
TIA1	p53	0.019	0.248	Moderate
CD20	p53	0.076	0.188	Weak
CD8	CD44s	0.086	0.182	Weak
TIA1	CD44s	0.087	0.180	Weak
CD4	CD95/Fas	0.089	0.178	Weak

Legend: TIL—tumor-infiltrating lymphocytes.

## Data Availability

The data presented in this study are available on request from the corresponding author.

## Supplementary Materials

The following are available online at https://www.mdpi.com/article/10.3390/cancers13236075/s1, Figure S1: Immunohistochemistry representative images (400× amplification) of positive samples from OST patients for TIL infiltration and tumor markers expression, Figure S2: Overall survival and progression-free survival functions calculated for the OST patients enrolled in the study, Figure S3: Time to the occurrence of metastasis regarding CD44v6 and CD44s expression calculated for the OST patients enrolled in the study, Figure S4: Overall and progression-free survival regarding the combination of positive and negative samples for CD4+ cells infiltration and CD44s expression calculated for the OST patients enrolled in the study, Figure S5: Overall survival and progression-free survival calculated for OST patients according to sex, age, tumor location and the use of neoadjuvant chemotherapy, Table S1: Primary specifications of: antibodies, staining, and laboratorial techniques applied to the tumor tissue samples from OST patients.
