# Supplementary material for: Tumor-Infiltrating Lymphocytes and Cancer Markers in Osteosarcoma: Influence on Patient Survival
Source: Cancers (Basel). 2021 Dec 2;13(23):6075. doi: 10.3390/cancers13236075 (PMC8656728; doi:10.3390/cancers13236075)
Supplement: Supplementary file 1 [file cancers-13-06075-s001.zip › cancers-1459383-supplementary.pdf]

cancers

MDPI

Supplementary Materials: Tumor-infiltrating lymphocytes and cancer markers in osteosarcoma: influence on patient survival

José Manuel Casanova, Jani-Sofia Almeida, John David Reith, Luana Madalena Sousa, Ruben Fonseca, Paulo Freitas-Tavares, Manuel Santos-Rosa, Paulo Rodrigues-Santos

Table S1. Primary specifications of: antibodies, staining, and laboratorial techniques applied to the tumor tissue samples from OST patients.

Antibody	Class	Target	Isotype	Clone	Reference	Brand	Antigenic recovery	Staining	Ventana Kit	Control tissue	Dil.	Staining
**Anti-human CD3**	Mono	T cells.	Rabbit		A 452	DAKO corporation; CA, USA	Microwave, citrate buffer	Ventana	DAB detection Kit	Lymph node	01:50	Membrane
**Anti-human CD4**	Mono	CD4 T cells	Rat / IgG1	1F6	NCI-CD4-IF6	Novocastra Laboratories Ltd; NCL, UK	Microwave, EDTA solution	Ventana	DAB high Intensity amplification Kit	Lymph node	01:20	Membrane
**Anti-human CD8**	Mono	CD8 T cells	Rat / IgG1	C8/144B	M-7103	DAKO corporation; CA, USA	Microwave, citrate buffer	Ventana	Amplification Kit	Lymph node	01:20	Membrane
**Anti-human TIA1**	Mono	NK and CD8 T cells	Rat / IgG1	TIA-1	PN 6604593	Coulter Corporation Ltd; CA, USA	Microwave, citrate buffer	Ventana	Amplification Kit	Lymph node	0.146	Membrane (granules)
**Anti-human CD20**	Mono	B cells	Rat / IgG2a	L26	M 755	DAKO corporation; CA, USA	Microwave, citrate buffer	Ventana	DAB detection Kit	Lymph node	0.111	Membrane
**Anti-human CD95/Fas**	Mono	Apoptosis mediator	Rat / IgG1	APO-1	M3553	DAKO corporation; CA, USA	Protease 2	Manual		Lymph node / tonsil	01:10	Membrane or cytoplasm
**Anti-human Fas-L**	Poly	Apoptosis mediator.	Rabbit	N-20	sc-834	Santa Cruz Biotechnology; CA, USA	Microwave, citrate buffer	Ventana	DAB detection Kit	Colon adenocarcinoma	01:25	Membrane or cytoplasm
**Anti-human CD44s**	Mono	Migration and cell signaling.	Rat / IgG1	DF 1485	M7082	DAKO corporation; CA, USA	Microwave, citrate buffer	Manual		Lymph node / tonsil	0.097	Membrane
**Anti-human CD44v6**	Mono	Metastatic processes.	Rat / IgG1	VVF7	NCL-CD44v6	Novocastra Laboratories Ltd; NCL, UK	Microwave, citrate buffer	Ventana	DAB high Intensity amplification Kit	Colon adenocarcinoma with lymphatic metastases	01:50	Membrane
**Anti-human p53**	Mono	Apoptosis and cell cycle.	Rat / IgG2b	DO-7	M7001	DAKO corporation; CA, USA	Microwave, citrate buffer	Ventana	Amplification Kit	Breast adenocarcinoma	01:50	Nuclear
**Anti-human P-glycoprotein**	Mono	Drug resistance.	Cow / IgG1	JBS-1	YM-9001	Accurate Chemical & Scientific Corporation; NY, USA.		Ventana	Amplification Kit	Kidney tissue	01:50	Membrane and Golgi complex

Legend: Mono – Monoclonal; Poly – Polyclonal; Dil. – Dilution.

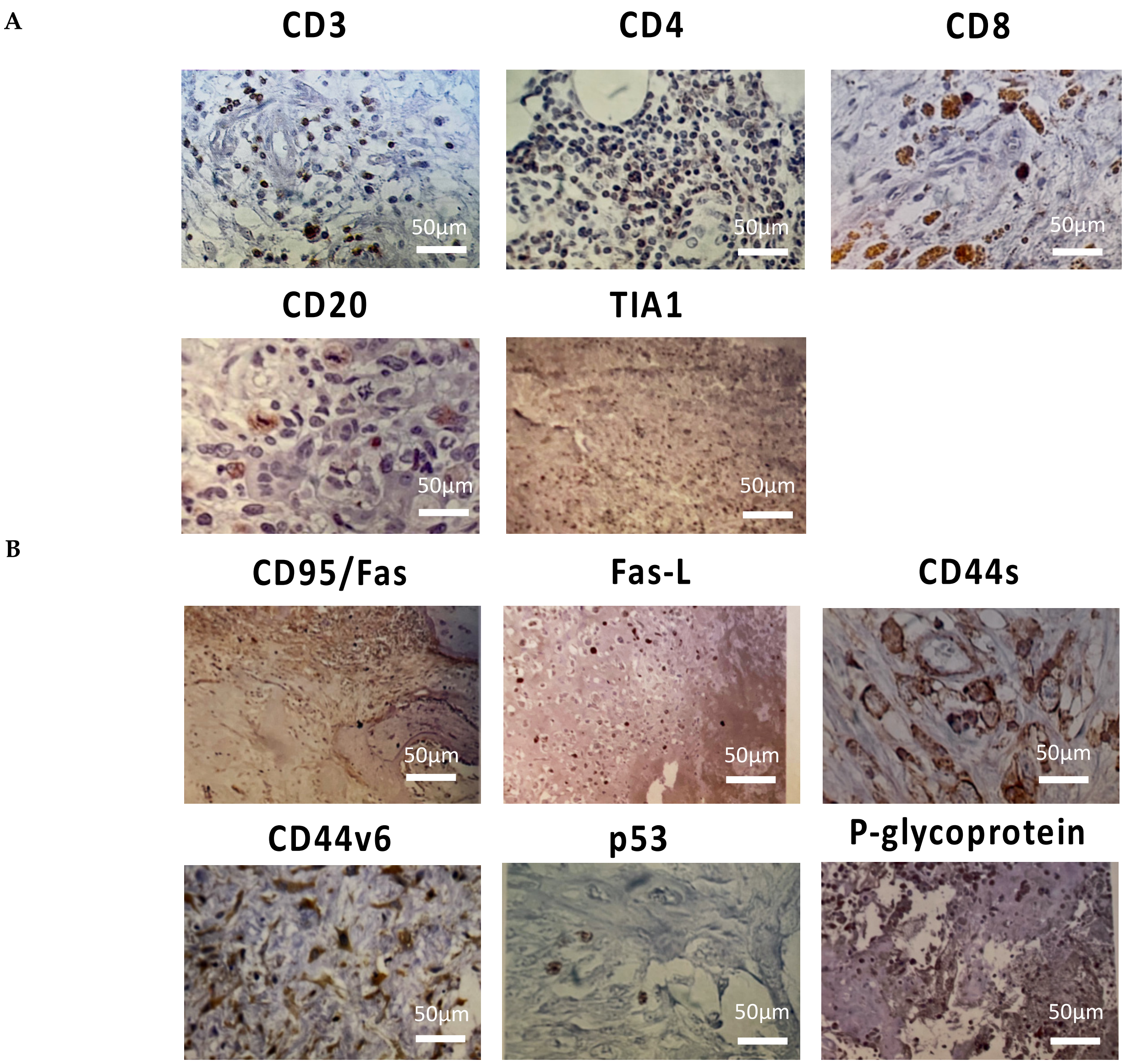

Figure S1. Immunohistochemistry representative images (400× amplification) of positive samples from OST patients for TIL infiltration and tumor markers expression. Scale bar, 50 µm.

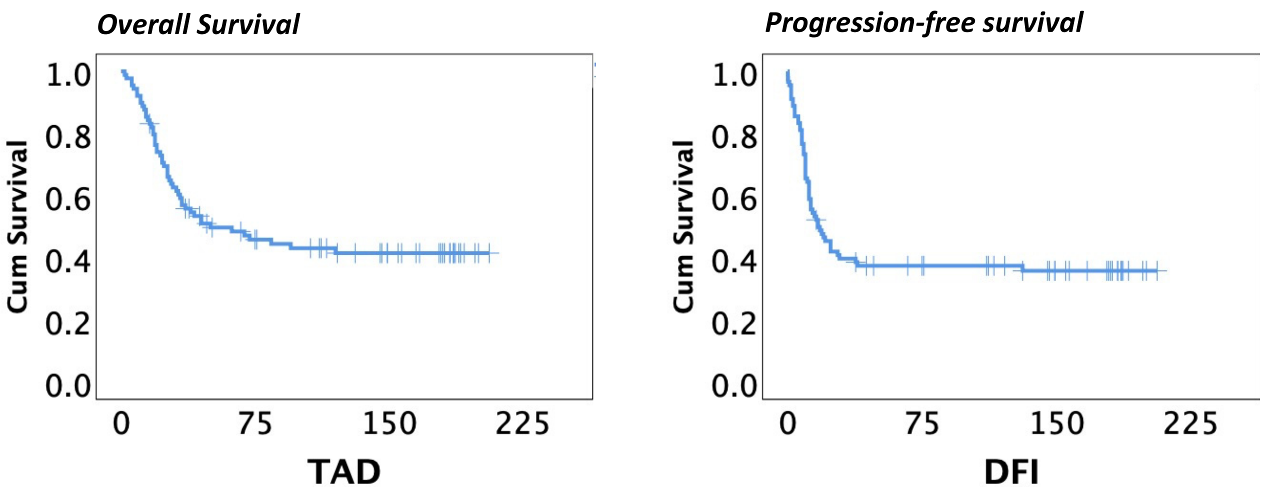

Figure S2. Overall survival and progression-free survival functions calculated for the OST patients enrolled in the study. TAD – Time after diagnosis (months); DFI – Disease-free interval (months).

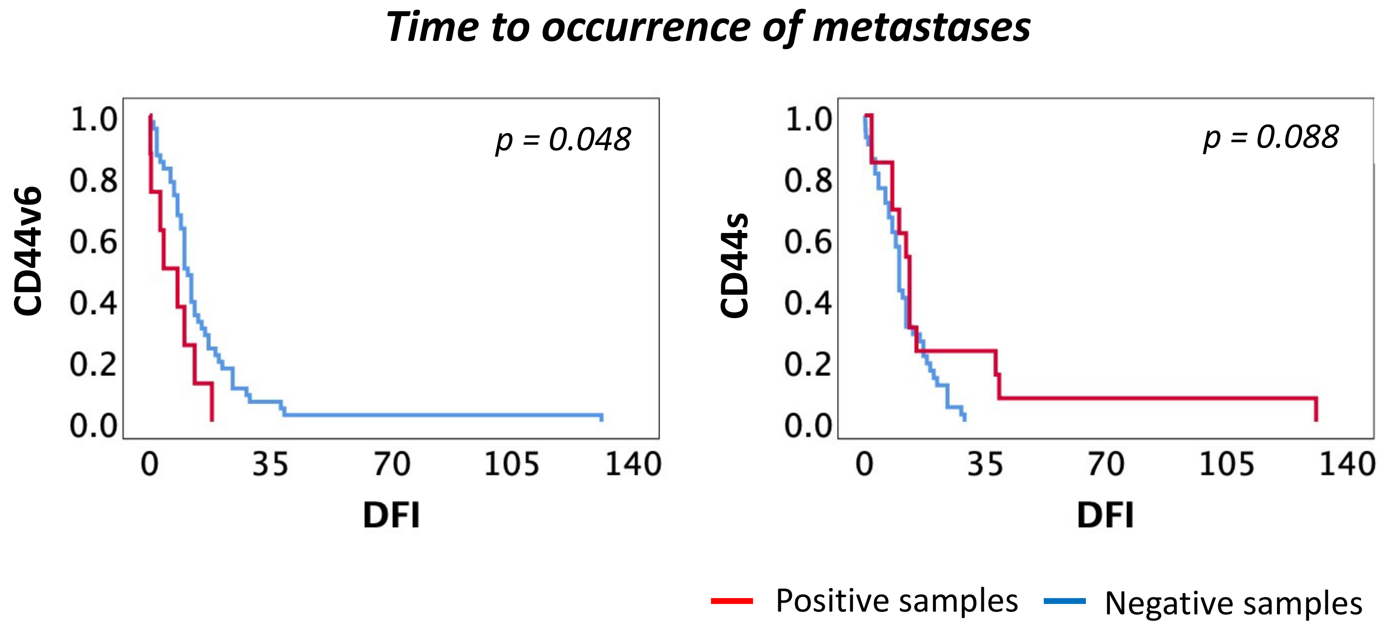

Figure S3. Time to the occurrence of metastasis regarding CD44v6 and CD44s expression calculated for the OST patients enrolled in the study. DFI – Disease-free interval (months).

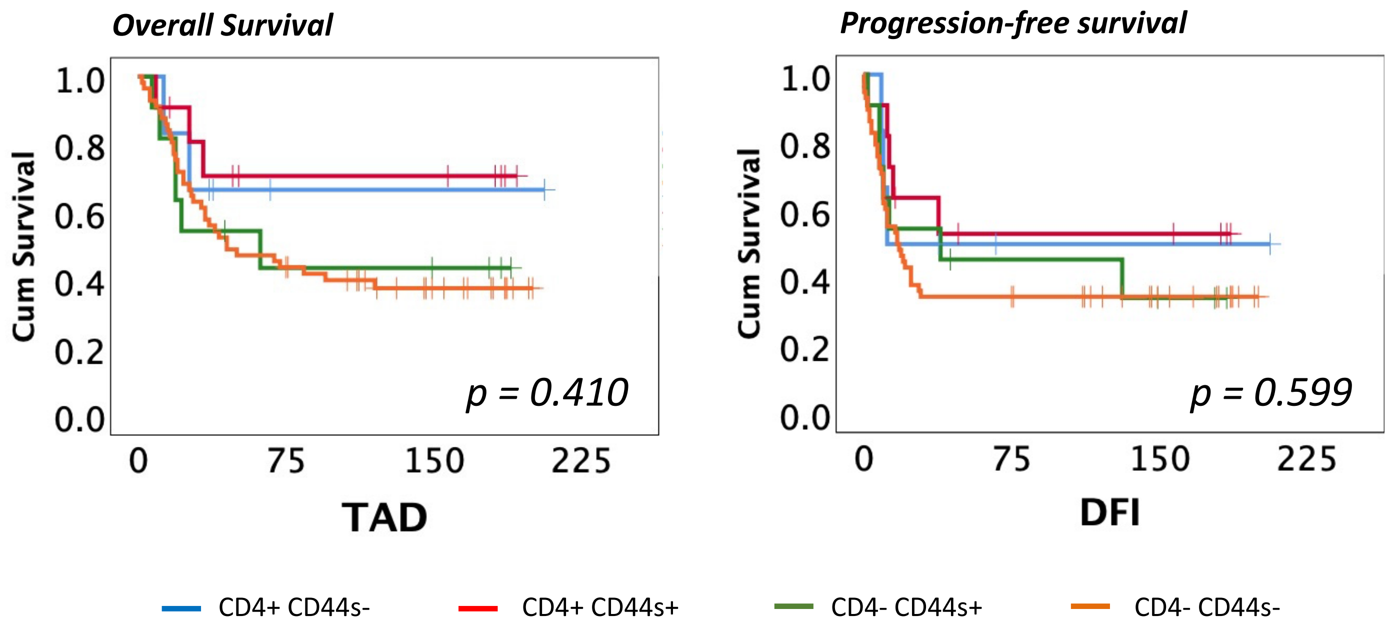

Figure S4. Overall and progression-free survival regarding the combination of positive and negative samples for CD4+ cells infiltration and CD44s expression calculated for the OST patients enrolled in the study. TAD – Time after diagnosis (months); DFI – Disease-free interval (months).

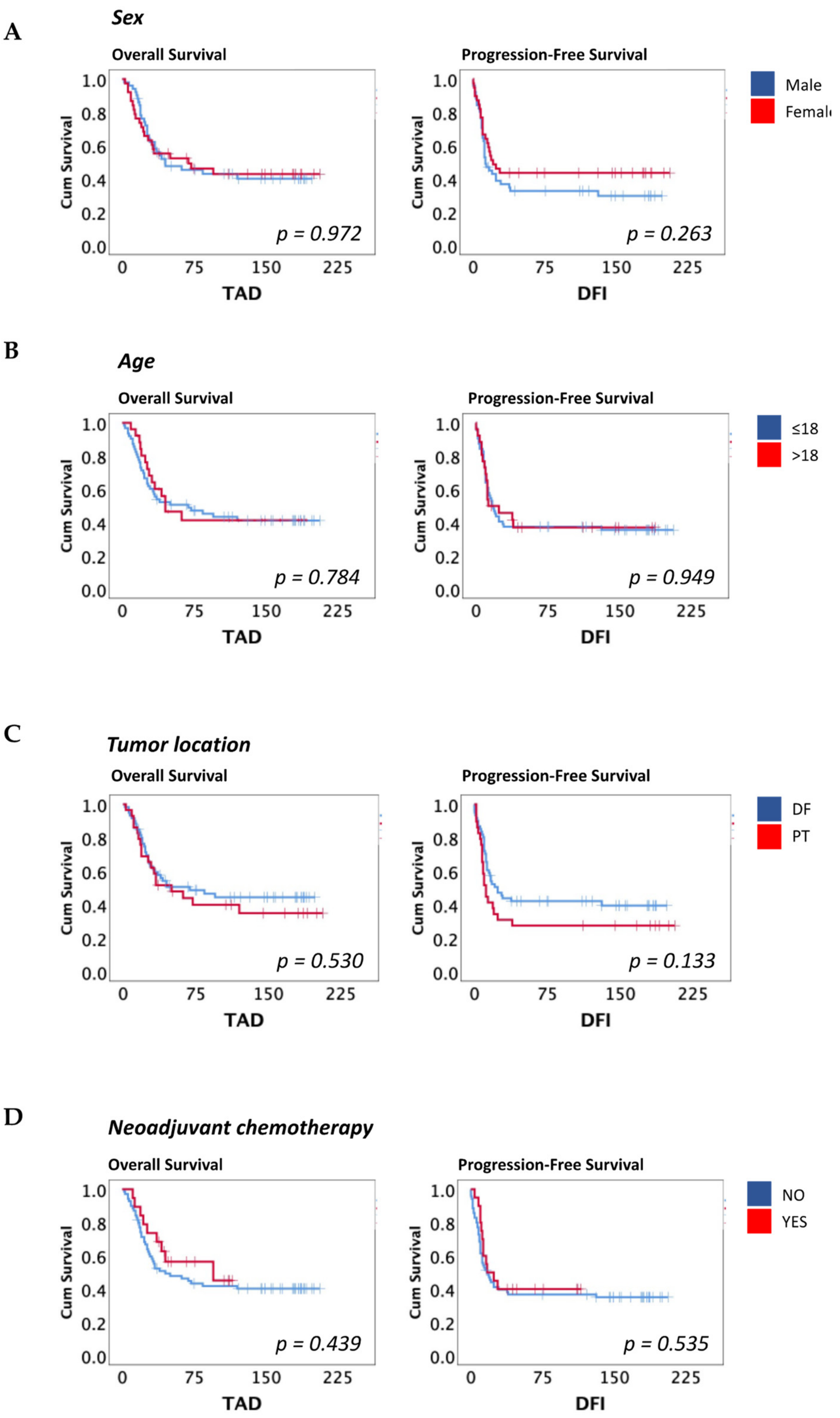

Figure S5. Overall survival and progression-free survival calculated for OST patients according to sex, age, tumor location and the use of neoadjuvant chemotherapy. TAD – Time after diagnosis (months); DFI – Disease-free interval (months).
